# Effects of a Polymorphism of the Neuronal Amino Acid Transporter *SLC6A15* Gene on Structural Integrity of White Matter Tracts in Major Depressive Disorder

**DOI:** 10.1371/journal.pone.0164301

**Published:** 2016-10-10

**Authors:** Sunyoung Choi, Kyu-Man Han, June Kang, Eunsoo Won, Hun Soo Chang, Woo Suk Tae, Kyu Ri Son, Su-Jin Kim, Min-Soo Lee, Byung-Joo Ham

**Affiliations:** 1 Department of Brain and Cognitive Engineering, Korea University, Seoul, Republic of Korea; 2 Department of Psychiatry, Korea University College of Medicine, Seoul, Republic of Korea; 3 Department of Biomedical Sciences, Korea University College of Medicine, Seoul, South Korea; 4 Department of Medical Bioscience, Graduate school, Soonchunhyang University, Bucheon, South Korea; 5 Brain Convergence Research Center, Korea University Anam Hospital, Seoul, South Korea; 6 Department of Radiology, Anam Hospital, Korea University College of Medicine, Seoul, Republic of Korea; 7 Department of Emergency Medicine, College of Medicine, Korea University, Seoul, Republic of Korea; University of Texas Health Science Center at San Antonio Cancer Therapy and Research Center at Houston, UNITED STATES

## Abstract

**Background:**

The *SLC6A15* gene has been identified as a novel candidate gene for major depressive disorder (MDD). It is presumed to be involved in the pathophysiology of MDD through regulation of glutamate transmission in the brain. However, the involvement of this gene in microstructural changes in white matter (WM) tracts remains unclear. We aimed to investigate the influence of a polymorphism of this gene (rs1545853) on the structural integrity of WM tracts in the cortico-limbic network.

**Methods:**

Eighty-six patients with MDD and 64 healthy controls underwent T1-weighted structural magnetic resonance imaging, including diffusion tensor imaging (DTI), and genotype analysis. We selected the genu of the corpus callosum, the uncinate fasciculus, cingulum, and fornix as regions of interest, and extracted fractional anisotropy (FA) values using the FMRIB Diffusion Toolbox software.

**Results:**

FA values for the left parahippocampal cingulum (PHC) was significantly reduced in the patients with MDD compared to healthy control participants (p = 0.004). We also found that MDD patients with the A allele showed reduced FA values for the left PHC than did healthy controls with the A allele (p = 0.012). There was no significant difference in the FA value of left PHC for the comparison between the G homozygotes of MDD and healthy control group.

**Conclusions:**

We observed an association between the risk allele of the *SLC6A15* gene rs1545843 and the WM integrity of the PHC in MDD patients, which is known to play an important role in the neural circuit involved in emotion processing.

## Introduction

Major depressive disorder (MDD) is one of the leading causes of disability and poses a great socioeconomic burden worldwide [1, 2]. Characterization of neurobiological mechanisms underlying MDD is a fundamental aim and goal of biological psychiatry and related fields of neuroscience research. In recent decades, numerous studies have suggested that the interaction of susceptible genotype and environmental factors, such as childhood adversity or stressful life events, play an important role in the pathophysiology of MDD [3, 4]. The gene-by-environment interaction is known to lead to biochemical disturbance, dysfunctional neural networks, and structural alteration of the brain in MDD patients [5]. Recent developments in techniques of neuroimaging analysis have encouraged novel methodological approaches using structural changes in the brain as an endophenotype in genetic studies of MDD [6]. Numerous studies have found that several candidate genetic polymorphisms, including serotonin transporter-lined polymorphic region (*5-HTTLPR)*, brain-derived neurotrophic factor (*BDNF*) Val66Met, tryptophan hydroxylase-2 (*TPH-2*), catechol-o-methyltransferase (*COMT*) Val158Met, serotonin receptor 1A gene (*HTR1A*), and monoamine oxidase A gene (*MAOA*) modulate morphologic brain changes in MDD patients [6, 7].

The *SLC6A15* gene, which belongs to the solute carrier 6 (SLC6) family, was recently identified by a genome-wide association study (GWAS) as a candidate gene for MDD [8]. This gene encodes a sodium-dependent branched-chain amino acid transporter, which is highly expressed in neurons in several brain regions [9]. Because proline has a high affinity for *SLC6A15* and is a precursor of glutamate synthesis, the *SLC6A15* gene is thought to be involved in the pathophysiology of the regulation of glutamate transmission in the brain in MDD [8]. In a very recent animal study, a *SLC6A15*-knockout mouse showed anxiety- and depressive-like behaviors under chronic social stress compared to its wildtype littermate, and the expression of the glutamate receptor (GluR1) has been shown to be regulated by SLC6A15 expression [10]. The results of another animal study have suggested that *SLC6A15* affects the hippocampal tissue levels of proline and glutamate/glutamine and that these neurochemical alterations are associated with behavioral abnormalities in sensorimotor gating, which is a translational endophenotype that has been linked to many psychiatric disorders [11]. In an investigation of the hypothalamic-pituitary-adrenocortical (HPA) axis abnormalities in MDD, Schuhmacher et al. have reported that one single nucleotide polymorphism (SNP) of *SLC6A15* (rs1545843) is associated with alterations in HPA axis activity, memory, and attention in patients with MDD [12]. The results of these studies indicate that the *SLC6A15* gene affects the predisposition to depression through alterations in glutamate neurotransmission in the hippocampus, HPA axis activity, and stress susceptibility.

The recently developed magnetic resonance imaging (MRI) technique, diffusion tensor imaging (DTI), has revealed microstructural changes in the brain [13]. DTI, which measures the direction and magnitude of motility of water molecules in brain tissue, has made it possible to determine both the orientation and diffusion characteristics of white matter (WM) tracts [14]. The most commonly used diffusion measurement, fractional anisotropy (FA), is represented as a scalar value between 0 and 1. Altered FA value without gross pathologic findings in the brain reflects disease-related microstructural change in WM tracts [15]. Recent DTI studies on patients with MDD have found alterations in the integrity of WM tracts that are involved in the cortico-limbic circuit, including the genu of the corpus callosum [16], cingulum [17], uncinate fasciculus [18], and fornix [19]. A recent neuroimaging study has reported reduced FA values in the inferior fronto-occipital fasciculus, uncinate fasciculus, and anterior thalamic radiation in patients with MDD and a negative correlation of the FA values in these WM tracts with the serum cortisol levels in the patients [20]. Reduced FA values in the anterior limb of the internal capsule in MDD and its negative correlation with depression severity have also been reported by another study [21].

In regards with SLC6A15 and structural neuroimaging findings, Kohli and colleagues reported that a risk allele of the rs1545843, of the *SLC6A15* gene located on ch12q21.31, is associated with alterations in hippocampal volume and neuronal integrity [8]. They also suggested, based on an animal model experiment, that the risk allele is associated with down-regulated expression of this gene in hippocampus and structural change of hippocampus under conditions of chronic stress. There have been very few studies on the *SLC6A15* gene and brain morphology changes in MDD patients. Apart from the study by Kohli et al. [8], only one preliminary study has been reported. This was a voxel-based morphometric study of 278 healthy controls [22], that indicated that rs1545843 is associated with reduced gray matter volume in the cingulate gyrus. Although there has been a recent increase in attention to DTI studies in the field of biological psychiatry, there have been no studies investigating the association between the *SLC6A15* gene and WM tracts. Thus, it remains unclear how this gene affects microstructural changes in WM tracts in MDD patients.

In this study, we analyzed DTI of MDD patients to investigate whether there is an interactive effect between the rs1545843 polymorphism of the *SLC6A15* gene and MDD diagnosis on microstructural change of WM tracts. Our *a priori* hypothesis was that the risk allele affects the integrity of WM tracts involved in the cortico-limbic network of emotion regulation including the genu of corpus callosum (GCC), fornix, cingulum, and uncinate fasciculus in MDD patients.

## Materials and Methods

### Participants

We recruited a total of 86 patients diagnosed with MDD from the outpatient psychiatric clinic of Korea University Anam Hospital, located in Seoul, South Korea. Trained psychiatrists examined all of the MDD patients with the Structured Clinical Interview for DSM-IV Axis I disorders (SCID-I). The exclusion criteria were (1) presumptive primary comorbid diagnosis of any other major psychiatric illness (based on DSM-IV criteria) on Axis I or Axis II, including anxiety disorders and substance abuse or dependence within the last 6 months; (2) suffering from serious or unstable medical illness; (3) primary neurological illness, such as cerebrovascular disease, Parkinson’s disease, and epilepsy, and (4) any contraindication for MRI. The duration of illness for MDD was assessed in an interview with the life-chart methodology.

Sixty-four healthy people without histories of psychiatric problems were recruited for the control group by advertisements in the community. The age of participants in both groups ranged from 21 to 69 years. All participants in both groups were right-handed, as revealed by the Edinburgh Handedness Test [23]. The severity of depressive symptoms was evaluated in both participant groups on the same day as the magnetic resonance image (MRI) scan with the 17-item Hamilton Depression Rating Scale (HDRS) [24]. During the study, 46 patients were undergoing antidepressant treatment, and 40 patients were drug-naïve. Eighteen patients received selective serotonin reuptake inhibitor (SSRI), 4 patients received serotonin and norepinephrine reuptake inhibitor (SNRI), 4 patients received norepinephrine-dopamine reuptake inhibitor (NDRI), and 3 patients received noradrenergic and specific serotonergic antidepressant (NaSSA). Seventeen of the patients received combinations of two or more types of antidepressants. Data for 113 of the 150 participants (60 MDD patients and 53 healthy controls) were reported in our previous publication [25]. In accordance with the Declaration of Helsinki, all participants gave informed consent to participate in the study, and the study protocol was approved by the ethics committee of the hospital.

### Genotyping

The genomic DNA was extracted from peripheral venous blood from each participant under a protocol approved by the Ethics Committee of the Korea University Medical Center. The genotype of *SLC6A15* rs1545843 was analyzed according to a previously described protocol [12]. Polymerase chain reaction was performed using the following primers: forward primer 5’-TTGGAATGGGAAAAGGGAGTC-3’ and reverse primer 5’- GGTTGTCCTTACTTTCTGGTGAA-3’. The genotyping success rate was above 95%. The allele frequencies (G allele/A allele) are as follows: MDD patients 0.63/0.37, healthy control participants 0.75/0.25. The genotype was in Hardy-Weinberg equilibrium as shown in Table 1.

**Table 1:** Demographic and clinical characteristics of major depressive disorder patients and healthy controls.

Characteristic	MDD (n = 86)	Healthy Controls (n = 64)	p-value
Age (Years)	44.34 ± 12.34	41.69 ± 14.58	0.243
Sex, Female	68	43	0.101
Education (Years)	12.23 ± 4.59	13.43 ± 4.79	0.125
Family history of MDD, Yes	26	1	< 0.001
Duration of illness (Months)	42.48 ± 45.29		
HDRS scores	14.87 ± 8.17	2.08 ± 2.10	< 0.001
Drug-naïve patients	40 (46.5%)		
Number of past depressive episode			
0	37 (43.0%)		
1	24 (27.9%)		
2	6 (7.0%)		
3 or more	19 (22.1%)		
SLC6A15 genotype (rs1545843)			
GG	36	34	0.189^a^
AG	36	28	
AA	14	2	
HWE^b^	0.334	0.182	

Data are mean ± standard deviation in age, education years, duration of illness, and HDRS scores.

The P values for distribution of sex and family history of MDD were obtained by chi-square test.

^a^The P value was obtained by chi-square test on distribution of subjects with GG genotype and A allele carrier (AA or AG) in MDD and healthy control groups.

^b^The P value of Hardy-Weinberg equilibrium in each group.

The P values for comparison in age, education years, and HDRS scores were obtained using two sample t-tests.

Allele frequencies (G/A): MDD patients 0.63/0.37, HC subjects 0.75/0.25.

MDD, major depressive disorder; HC, healthy controls; HDRS, Hamilton Depression Rating Scale; HWE, Hardy-Weinberg equilibrium.

### MRI data acquisition

Diffusion data were acquired on a Siemens Trio whole-body imaging system (Siemens Medical Systems, Erlangen, Germany). Diffusion tensor images were acquired using an echo-planar imaging sequence with the following parameters: repetition time (TR) 6300 ms; echo time (TE) 84 ms; field of view (FOV) 230 mm; 128 × 128 matrix; 3-mm slice thickness with no gap; voxel size 1.8 mm × 1.8 mm × 3.0 mm; diffusion directions = 20; number of slices = 50; b-values 0 and 600 s/mm^2^; acceleration factor (iPAT-GRAPPA) 2 with 38 reference lines for phase encoding direction and 6/8-phase partial Fourier.

### Image processing

**Fig 1:**
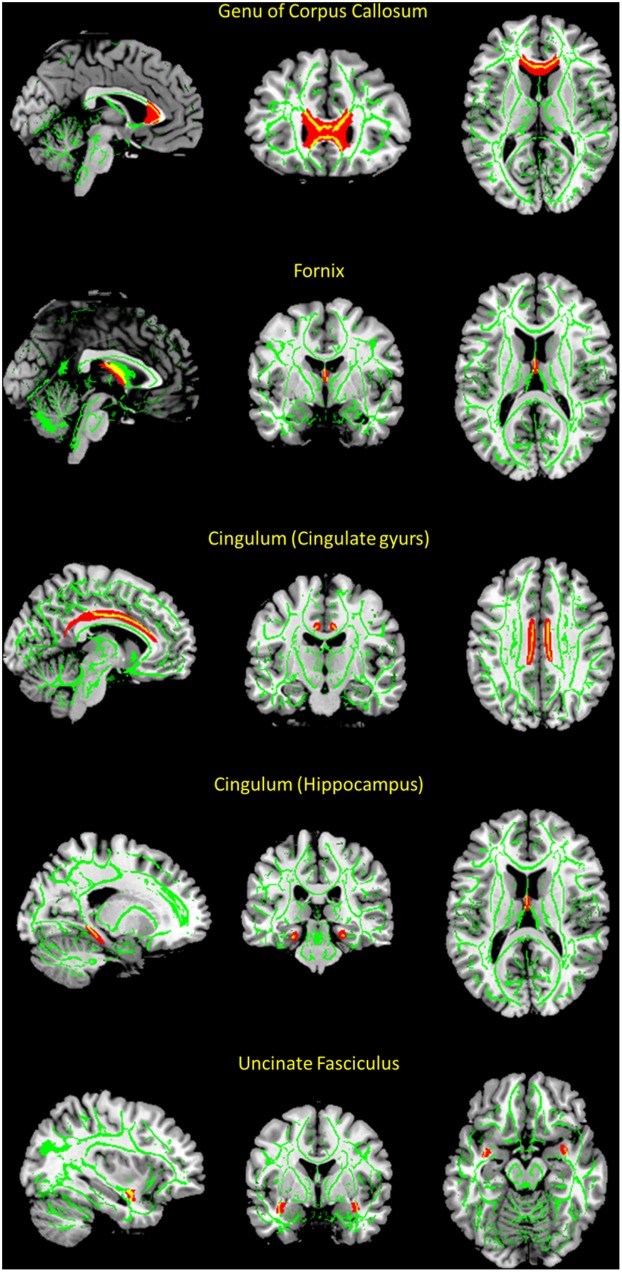
White matter regions of interests (ROIs) from JHU white matter atlas and skeletonized white matter tracts derived from tract-based spatial statics (TBSS). Each ROI within JHU white matter atlas is represented in red color, and each skeletonized white matter tract is represented in green color. Fractional anisotropy values are extracted from the overlapping regions of ROIs within JHU atlas and skeletonized white matter tracts, which are represented in yellow color. (Red color = ROIs within JHU atlas; green color = skeletonized white matter tract; yellow color = overlapping regions of ROIs and skeletonized white matter tracts)

To investigate the structural integrity of WM tracts in the cortico-limbic circuit, we selected the genu of the corpus callosum, uncinate fasciculus, cingulum (cingulate gyrus and parahippocampal region, respectively), and fornix as regions of interest (ROIs). These WM tracts were selected based on previous studies aimed at examining the microstructural change of WM tracts in the cortico-limbic circuit in patients with MDD [7, 15, 26]. Eight WM tracts in the ROIs were selected with the Johns Hopkins University (JHU) WM tractography atlas, which is included in the FMRIB Diffusion Toolbox (FDT) [27], as shown in Fig 1.

We used the average FA values of selected WM tracts as a measure of structural integrity of the WM tracts. The FA values were calculated with the automated procedure of the FDT software [28]. The extraction of FA values in WM tracts was performed according to a previously described protocol of ENIGMA-DTI working group [29]. Briefly, we first corrected for eddy current distortions and head movement in the raw DTI data. Second, using the Brain Extraction Tool (BET) with a brain extraction factor of 0.3, non-brain voxels were excluded. Third, the diffusion tensor models were fitted to the corrected FA data, and individual FA images were produced. The extraction of individual FA values in each WM tract was performed in the following steps: First, we transformed individual DTI and brain-extracted anatomical images into standard Montreal Neurological Institute (MNI) space using the FMRIB Nonlinear Image Registration Tool (FNIRT). Second, we performed a mapping from standard MNI space onto individual DTI space; this mapping was then used in the transformation of each template ROI into individual-specific DTI space. Finally, we extracted the average FA values of the ROIs in the individual FA images with the individual DTI space ROIs. The extracted average FA values of the ROIs were used in the main-analysis. The normality of the distribution of the FA values in the ROIs in each group was tested with the Kolmogorov-Smirnov test, and significant deviations from normal distributions were not observed (all, p > 0.05).

In order to examine the effects of any interactions of genotype and diagnosis in the WM tracts other than those in the ROIs of this study, we performed an additional explorative whole-brain analysis after the ROI analysis. Forty-two WM tracts in the whole brain were selected with the Johns Hopkins University (JHU) atlas. A list of the 42 WM tracts in the brain is presented in S1 Table.

### Statistical analyses

In the main analysis, we performed a two-way ANCOVA with the independent variables of the two diagnostic groups [patients with MDD vs. healthy controls (HCs)] and the *SLC6A15* rs1545854 genotype; the dependent variable of the extracted FA values of the 8 WM tracts of the ROIs; and the covariates of age, sex, and the demeaned value of the total intracranial cavity volume (TICV) to investigate whether there was a diagnosis-by-genotype interaction. We included the TICV as a covariate because we observed correlations between the TICV and FA values of the WM tracts of several ROIs. We performed genotype-based analysis with the dominant model and compared the participants with the GG genotype to the A allele carriers (AA or AG), as differences in these populations have been reported in previous studies on *SLC6A15* rs1545843 and depression [8, 12]. For the secondary analysis, we performed pair-wise comparisons of the FA values of the ROIs by using one-way ANCOVA with the same covariates as those in the above analysis on the genotype/diagnosis subgroups: i.e., the MDD group with the A allele vs. HC group with the A allele, MDD group with the GG genotype vs. HC group with the GG genotype, MDD group with the A allele vs. MDD group with the GG genotype, and the HC group with the A allele vs. HC group with the GG genotype. In addition, we performed a whole-brain analysis of the 42 WM tracts and used the same statistical methods as those used in the main analysis to examine the effects of the genotype-by-diagnosis interaction in the WM tracts other than those in our ROIs. To correct for multiple comparisons, Bonferroni’s method was applied on the effects of diagnosis, genotype, and the diagnosis-by-genotype interaction with an alpha level of p < 0.00625 (0.05/8 comparisons: 8 WM tracts in both hemispheres) in the ROI analysis, and the False Discovery Rate (FDR) of Benjamini and Hochberg (1995) [30] was applied in the additional explorative whole-brain analysis (42 comparisons of the WM tracts of both hemispheres, q < 0.05). The differences in demographic and clinical characteristics of the patients with MDD and HC group were analyzed with t-tests for age, years of education, and HDRS scores. The distribution of sex, family history of MDD, and genotype were analyzed with a chi-square test. Statistical analyses were performed using SPSS version 12.0 (SPSS Inc., Chicago, IL, USA).

## Results

### Demographic and genotype characteristics

The age, sex distribution, education years, family history of MDD, duration of illness, HDRS scores, number of past depressive episodes, and allelic variants of the *SLC6A15* genotype of 86 patients with MDD and 64 healthy controls are shown in Table 1. There were no significant differences between the two groups in mean age, sex distribution, education years, and distribution of the genotype. There were significant differences between the two groups in HDRS scores and family history of MDD (both p < 0.001). The mean duration of illness in the patients with MDD was 42.03 months. Among the MDD patients, 40 participants were drug-naïve, and 46 participants were taking antidepressants.

### Comparison of FA values according to genotype and diagnosis

In the ROI analysis, the patients with MDD had lower FA values in the parahippocampal cingulum (PHC) compared to healthy control participants (F_(1, 149)_ = 8.698, p = 0.004, Cohen’s *f* = 0.247, 0.57 ± 0.06 in MDD vs. 0.60 ± 0.06 in HC) and this difference remained significant after the Bonferroni correction. There were no significant differences in FA values between the participants with the GG genotype and those with the A allele. We performed an analysis on the diagnosis (patients with MDD vs. healthy controls)-by-genotype (GG vs. A allele carriers) interaction effect on the integrity of WM tract ROIs to test our *a priori* hypothesis, and could not find significant diagnosis-by-genotype interaction in the ROI analysis. The details of the ROI analysis are described in Table 2.

**Table 2:** The differences of fractional anisotropy values in the regions of interest white matter tracts among groups determined by genotype and diagnosis.

WM tracts (ROIs)	MDD vs. HC				AG/AA vs. GG			Diagnosis x Genotype interaction		
	F	p	Cohen's *f*		F	p	Cohen's *f*	F	p	Cohen's *f*
Genu of corpus callosum	2.764	0.099	0.139		0.528	0.469	0.061	0.198	0.657	0.037
Fornix (Column and body)	2.102	0.149	0.121		2.612	0.108	0.135	0.133	0.716	0.030
**Left hemisphere**										
Cingulum (hippocampus)	8.698	0.004*	0.247	MDD < HC	0.127	0.722	0.030	0.221	0.639	0.039
Cingulum (cingulate gyrus)	0.156	0.694	0.033		0.052	0.820	0.019	0.143	0.706	0.032
Uncinate fasciculus	0.554	0.458	0.062		3.705	0.056	0.161	3.662	0.058	0.160
**Right hemisphere**										
Cingulum (hippocampus)	1.323	0.252	0.096		0.14	0.709	0.031	0.026	0.873	0.013
Cingulum (cingulate gyrus)	<0.001	0.990	0.001		0.105	0.747	0.027	0.252	0.616	0.042
Uncinate fasciculus	0.113	0.737	0.028		0.136	0.713	0.031	0.658	0.419	0.068

The F and P values and Cohen's *f* were obtained using two-way analysis of covariance (ANCOVA) adjusted for age, sex, and total intracranial cavity volume as covariates.

Bonferroni correction was applied: 8 comparisons, corrected p-value = (0.05/8) = 0.00625.

*Regions that remained significant after Bonferroni correction are marked with an asterisk.

MDD, major depressive disorder; HC, healthy controls; Diagnosis x Genotype interaction, the diagnosis (MDD patients vs. healthy controls)-by-genotype (GG vs. AA/AG) interaction.

**Fig 2:**
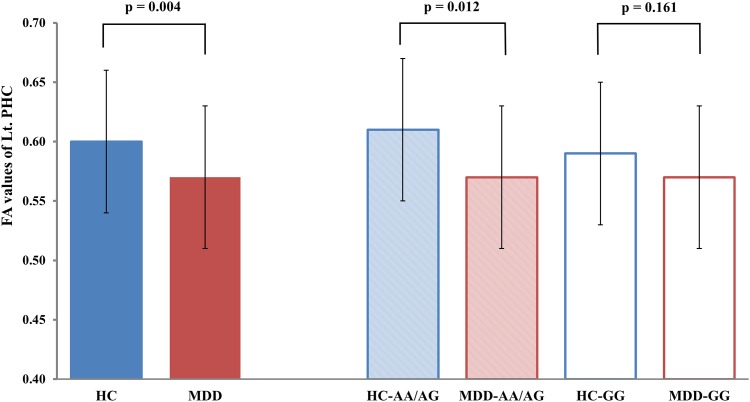
Comparison of the fractional anisotropy (FA) values of the left parahippocampal cingulum (PHC) among groups determined by genotype and diagnosis. The P values of comparisons among the groups were obtained using an analysis of covariance (ANCOVA) adjusted for age, sex, and total intracranial cavity volume as covariates. (HC, n = 64; MDD, n = 86; HC-AG/GG, n = 30; MDD-AG/GG, n = 50; MDD-GG, n = 36; HC-GG, n = 34; MDD-AA/AG, patients with MDD and an AA or AG genotype on rs1545843; MDD-GG, patients with MDD and the GG genotype on rs1545843; HC-AA/AG, healthy controls with the AA or AG genotype on rs1545843; HC-GG, healthy controls with the GG genotype on rs1545843; Blue and red color represent healthy control group and MDD group, respectively. Error bars represent standard deviation of FA value)

We also performed secondary pair-wise comparisons of the WM tracts in the ROIs by comparing the FA values for each region according to diagnosis and genotype. In the participants with the A allele, the patients with MDD had reduced FA values in the left PHC than the healthy controls (F_(1,79)_ = 6.651, p = 0.012, Cohen’s *f* = 0.298, 0.57 ± 0.06 in MDD vs. 0.61 ± 0.06 in HC), while, for the GG genotype group, diagnosis did not influence the FA values in the left PHC (p > 0.1). In the healthy control group, healthy controls with A allele had greater FA values in the left uncinate fasciculus (UF) than those with the GG genotype (F_(1,69)_ = 6.940, p = 0.011, Cohen’s *f* = 0.343, 0.63 ± 0.06 in A allele carrier vs. 0.59 ± 0.06 in G homozygotes). The details of the results are shown in Table 3 and Fig 2. In order to examine the potential effects of medication on the FA values, we compared the FA values of the ROIs between medicated (n = 46) and drug-naïve patients (n = 40) by using one-way ANCOVA that was adjusted for age, gender, TICV, HDRS score, and duration of illness, and no significant differences were found between any of the two subgroups (all, p > 0.1, except for the fornix, p = 0.067).

**Table 3:** Pair-wise comparisons of the fractional anisotropy values of region-of-interest white matter tracts among the groups determined by genotype and diagnosis.

**WM tract**	**FA values**					**FA values**				
	**MDD-AA/AG (n = 50)**	**HC-AA/AG (n = 30)**	**F**	**p**	**Cohen's *f***	**MDD-GG (n = 36)**	**HC-GG (n = 34)**	**F**	**p**	**Cohen's *f***
Genu of corpus callosum	0.69 ± 0.04	0.70 ± 0.04	1.639	0.204	0.148	0.69 ± 0.04	0.70 ± 0.03	0.812	0.371	0.112
Fornix (Column and body)	0.50 ± 0.09	0.52 ± 0.07	1.854	0.177	0.157	0.49 ± 0.08	0.51 ± 0.07	0.635	0.428	0.099
**Left hemisphere**										
Cingulum (hippocampus)	0.57 ± 0.06	0.61 ± 0.06	6.651	0.012*	0.298	0.57 ± 0.06	0.59 ± 0.06	2.012	0.161	0.176
Cingulum (cingulate gyrus)	0.62 ± 0.06	0.62 ± 0.03	0.006	0.940	0.009	0.63 ± 0.06	0.62 ± 0.04	0.433	0.513	0.082
Uncinate fasciculus	0.61 ± 0.08	0.63 ± 0.06	0.929	0.338	0.111	0.61 ± 0.07	0.59 ± 0.06	2.965	0.090	0.214
**Right hemisphere**										
Cingulum (hippocampus)	0.59 ± 0.05	0.60 ± 0.05	1.137	0.290	0.123	0.59 ± 0.05	0.60 ± 0.05	0.734	0.395	0.106
Cingulum (cingulate gyrus)	0.60 ± 0.06	0.61 ± 0.04	0.225	0.637	0.055	0.61 ± 0.07	0.61 ± 0.05	0.022	0.883	0.018
Uncinate fasciculus	0.60 ± 0.06	0.61 ± 0.06	0.642	0.425	0.093	0.61 ± 0.06	0.61 ± 0.06	0.082	0.776	0.035
**WM tract**	**MDD-AA/AG (n = 50)**	**MDD-GG (n = 36)**	**F**	**p**	**Cohen's *f***	**HC-AA/AG (n = 30)**	**HC-GG (n = 34)**	**F**	**p**	**Cohen's *f***
Genu of corpus callosum	0.69 ± 0.04	0.69 ± 0.04	0.023	0.880	0.017	0.70 ± 0.04	0.70 ± 0.03	1.797	0.185	0.175
Fornix (Column and body)	0.50 ± 0.09	0.49 ± 0.08	0.659	0.419	0.090	0.52 ± 0.07	0.51 ± 0.07	2.718	0.105	0.215
**Left hemisphere**										
Cingulum (hippocampus)	0.57 ± 0.06	0.57 ± 0.06	<0.001	0.991	0.001	0.61 ± 0.06	0.59 ± 0.06	0.122	0.729	0.045
Cingulum (cingulate gyrus)	0.62 ± 0.06	0.63 ± 0.06	0.155	0.695	0.044	0.62 ± 0.03	0.62 ± 0.04	0.014	0.906	0.015
Uncinate fasciculus	0.61 ± 0.08	0.61 ± 0.07	<0.001	0.986	0.002	0.63 ± 0.06	0.59 ± 0.06	6.940	0.011*	0.343
**Right hemisphere**										
Cingulum (hippocampus)	0.59 ± 0.05	0.59 ± 0.05	0.177	0.675	0.047	0.60 ± 0.05	0.60 ± 0.05	0.013	0.908	0.015
Cingulum (cingulate gyrus)	0.60 ± 0.06	0.61 ± 0.07	0.029	0.865	0.019	0.61 ± 0.04	0.61 ± 0.05	0.405	0.527	0.083
Uncinate fasciculus	0.60 ± 0.06	0.61 ± 0.06	0.913	0.342	0.106	0.61 ± 0.06	0.61 ± 0.06	0.227	0.635	0.062

Data are mean ± standard deviation (values of fractional anisotropy).

The F and P values and Cohen's *f* were obtained using two-way analysis of covariance.

(ANCOVA) adjusted for age, sex, and total intracranial cavity volume as covariates.

*Significant differences (p < 0.05) are marked with an asterisk.

FA, fractional anisotropy; MDD, major depressive disorder; HC, healthy controls; MDD-AA/AG, patients with MDD with AA or AG genotype on rs1545843; MDD-GG, patients with MDD with GG genotype on rs1545843; HC-AG/GG, healthy controls with AG or GG genotype on rs1545843; HC-GG, healthy controls with GG genotype on rs1545843.

As an additional explorative analysis, we performed a whole-brain analysis with the same statistical methods as those used in the ROI analysis to investigate the diagnosis-by-genotype interaction. In the whole-brain analysis, we did not found any significant diagnosis-by-genotype interactions in the 42 WM tracts after correcting for multiple comparisons, and genotype did not influence the FA values of the 42 WM tracts (S2 Table).

### Correlation among the genotypes, FA values of the PHC and UF, and depression severity

We performed post-hoc analysis on the correlation between the FA values of the bilateral PHC and UF and the severity of depressive symptoms, measured by HDRS score, in our patients with MDD. Pearson’s partial correlation analysis adjusting for age, sex, and TICV did not demonstrate any significant correlation between HDRS score and FA values of the bilateral PHC and UF in the MDD group (all, p > 0.1).

We observed significantly higher HDRS scores in patients with MDD who had the A allele compared to patients with the GG genotype (12.94 ± 7.41 in the GG genotype vs. 16.26 ± 8.48 in the A allele carrier; p = 0.035; F = 4.593, Cohen’s *f* = 0.237). However, healthy control participants did not show a significant difference in HDRS scores by genotype (1.74 ± 1.83 in the GG genotype vs. 2.47 ± 2.35 in the A allele carrier; p > 0.1) in the analysis using an ANCOVA adjusting for age and sex.

## Discussion

In this study, we investigated the influence of *SLC6A15* rs1545843 on microstructural changes of WM tracts in corticolimbic circuits in patients with MDD. We could not find significant interactive effects of diagnosis of MDD and the genotype; however, in the secondary analysis, we found that MDD patients with the A allele had lower FA values in the left PHC than healthy controls with the A allele, while there was no significant difference in WM integrity of the left PHC between the G homozygotes of the MDD group and the healthy control group. We also observed that healthy control participants with the A allele had greater FA values for UF than G homozygous healthy controls. To our knowledge, this is the first study to use DTI to elucidate the relationship between the *SLC6A15* genotype and WM microstructural change in MDD patients.

Recent DTI studies have reported an association between structural integrity of frontolimbic WM pathways and a polymorphism of *BDNF* and the neurotrophic tyrosine kinase receptor type 2 (5, 7), *5-HTTLPR* [31], and *FKBP5* gene [32]. However, we did not find a significant diagnosis-by-genotype interaction in the FA values of the WM tracts of the ROIs. We only observed indirect evidence in the results of the secondary analysis that suggested the existence of the effects of an interaction of *SLC6A15* rs1545843 and MDD on the WM tracts. The results of the secondary pair-wise comparison analysis showed that diagnosis had different effects on the FA value in the left PHC in both genotype (GG genotype vs. A allele carrier) groups. In the A allele carrier group, the patients with MDD had significantly decreased FA value in that region. Otherwise, no significant differences were observed between the patients with MDD and HC group with GG genotype, as shown in Fig 2. Genotype exhibited different effects according to diagnosis. In the HC group, the A allele was associated with increased FA value in the left UF, while the patients with MDD had no changes in the FA values in that region. However, our observations of risk allele-specific microstructural changes in the WM tracts in the ROIs, including the PHC and UF, were tentative because of the lack of a significant diagnosis-by-genotype interaction in the main analysis. Further studies are required to elucidate the association between *SLC6A15* rs1545843 and WM tract integrity in patients with MDD.

The *SCL6A15* gene is assumed to be involved in glutamate transmission because of its contribution to transport of leucine and proline, which are known to be precursors of glutamate [12]. A recent GWAS reported that the *SLC6A15* variant rs1545843 is associated with MDD [8]. In the same study, the authors stated that compared to a stress-resistant mouse, a stress-susceptible mouse model showed lower *SLC6A15* mRNA levels, and that diagnosis and the rs1545843 genotype had interaction effects on total hippocampal gray matter and cornu ammonis volume [8]. Another study found an influence of rs1545843 on adrenocorticotropic hormone (ACTH) and cortisol levels in MDD patients, and reported an association between this polymorphism and cognitive functions such as memory and sustained attention in these patients [12]. Although the suggested role of this gene in the stress-susceptibility reported in animal model studies and possible associations with a predisposition to MDD was investigated in a recent GWAS [8], there is no clear explanation of the exact neurobiological role of this genetic polymorphism in the pathophysiologic process of MDD and related structural changes in specific brain regions. We cautiously presume that neuronal amino acid transport and synthesis of glutamate modulated by the *SLC6A15* gene facilitated the observed the risk allele-specific microstructural change in PHC and UF. Further studies on this issue are required.

The PHC, which is the infracallosal part of the cingulum and is proximal to the hippocampus, is known to be a main communication route between the cingulate cortex and the hippocampus [32]. The PHC plays a critical role in the integration of cognitive and emotional information, and disruption of this WM tract could result in dysfunction of emotional and cognitive processing [32]. In the perspectives of the Papez circuit, the PHC is a main component of this circuit, connects the hippocampus and prefrontal cortex via entorhinal and cingulate cortex, and is deeply involved in emotion regulation [17]. The PHC and hippocampus are also major components of the posterior hub in the default mode network (DMN), and dysfunction of DMN and related structural change in the PHC is deeply implicated in the predisposition to MDD [33]. Structural abnormalities in PHC is reported in previous DTI studies on post-traumatic stress disorder [34], bipolar disorder [35], schizophrenia [36], Alzheimer’s disease [18], mild cognitive impairment [37], and childhood abuse [38]. Jiang et al., in their atlas-based tract-specific quantification and voxel-based analysis, showed that first-episode, treatment-naïve patients with MDD had a lower FA value in the PHC compared to healthy control participants [17]. WM lesions in the PHC were also associated with greater depression severity after antidpressant treatment in patients with late-life depression [39]. In an imaging genetic study, Fani et al. found that the risk allele of *FKBP5* gene rs1360780 was associated with reduced FA value of the left PHC in 82 traumatized female civilians [34]. Considering the functional and structural connection of the PHC and hippocapus in emotion processing, risk allele of *SLC6A15* rs1545843-specific hippocampal and cornu amonis volume reduction in patients with MDD could be a supportive evidence of our result [8].

With regard to the greater FA value of the UF in healthy controls with A allele compared to those with GG genotype, Ugwu and colleagues, in their study on DTI of 46 patients with MDD and 46 healthy controls, suggested that participants with a history of childhood adversity showed greater FA value in the UF, while diagnosis of depression did not affect the FA value in the UF [40]. However, our participants were not evaluated with respect to their experiences of childhood adversity. It is still not well understood why FA value is increased, as opposed to reduced, and why FA values of healthy controls are affected, but not those of patients with MDD. Some explanations may account for our observation. The limbic-cortical dysregulation model of depression implies that pathophysiology of depression is based on the hyperactivity of emotion processing circuits, including the limbic region and disturbance of the normal top-down emotion control network operated by the prefrontal cortex [41, 42]. Steffens et al. found in their functional MRI study that structural integrity of the UF was positively correlated with resting state functional connectivity (rsFC) between ventrolateral prefrontal cortex and both amygdala and hippocampus [42]. They suggested that higher rsFC between prefrontal cortex and limbic region, which reflects dysregulated normal top-down emotion control, might be associated with the increased FA value of the UF in their study. In our study, we postulated that increased WM integrity of the UF in healthy controls with the A allele might implicate that the risk allele of *SLC6A15* rs1545843 affects structural integrity of the neural network involved in emotion regulation and that A allele could be a predisposing factor for emotion and mood dysregulation. Our observation that patients with MDD with the A allele had greater HDRS score than those with the GG genotype also supports our postulation that A allele might be a predisposing factor for MDD and related microstructural changes in the WM tract. However, we still could not explain clearly why the risk-allele specific WM integrity change of UF is only observed in the healthy control group, not in the MDD group. We cautiously presume that the increased FA value in the UF in healthy controls with genetic predisposing factor might be an intermediate neural phenotype reflecting the pre-clinical pathophysiologic change in WM tracts, and expect that further studies on this issue could reveal the neurobiological mechanism underpinning this finding.

A causal relationship between the structural integrity change of the PHC in the patients with A allele of rs1545843 and MDD, represented by a dysfunctional neural network of emotion processing, remained to be elucidated. One possible explanation is that disruption of WM integrity of the PHC influenced by genetic predisposition (i.e. *SLC6A15* gene rs1545843 A allele) may act as a trait factor that leads to dysregulated neural network of emotion processing and the depressive state. Another possible explanation is that emotional dysregulation and depressive mood could lead to microstructural WM abnormalities in the patients with genetic predisposing factor. There is an expectation that debate on whether brain structural change is a trait or state factor of MDD could be resolved by well-designed longitudinal imaging-genetic studies, including both structural and functional brain imaging. In addition, underlying neurobiological basis of this ‘genotype and diagnosis-specific effect’ of the *SLC6A15* gene on the microstructural change of the WM tract must be elucidated in future studies.

To our knowledge, this is the first study to integrate the *SLC6A15* genotype and WM microstructural changes in MDD patients using DTI; thus future studies should replicate and further explore our results. The sample size in our study is relatively large, compared to that in recent studies, which found an influence of genotype-by-diagnosis interaction on WM microstructural changes, by using genetic modality and DTI [43–45], and we presumed that this could ensure significant statistical power in our study. There are also several limitations to our study, which should be considered, along with its strong points. First, our main findings of risk-allele-specific WM integrity changes in the PHC and UF were totally dependent on the results of the secondary analysis. However, we did not observe significant diagnosis-by-genotype interactions in these WM tracts in the main analysis. Thus, our main findings indirectly suggested the existence of interaction effects of the *SLC6A15* genotype and MDD on the PHC and UF. We believe that further studies are required to obtain direct evidence for the interaction effects of the genotype and MDD on WM tract integrity. Furthermore, we performed secondary pair-wise comparisons to assess the effects of genotype in each diagnostic group and of diagnosis in each genotype group. The application of both types of analyses instead of only one might have increased the type-I error. Second, about half of our MDD patients (53.5%) were taking antidepressants. There have been several suggestions from DTI studies on MDD that antidepressant medications might have an influence on microstructural changes in WM tracts [46]; however, whether antidepressant treatment affects WM integrity is still a matter of ongoing research. In our study, there were no differences in FA values of ROI regions between drug-naïve MDD patients and those on antidepressant treatment, even when applying the same statistical method and threshold to the main results. Therefore, we concluded that antidepressants could not influence our main result of risk allele-specific reduction of FA value in left PHC of MDD patients. Third, we did not find any correlation of FA values in the PHC or UF with severity of depression, and could not suggest the association of microstructural change of these WM tracts with clinical characteristics of MDD. Future studies should investigate this issue. Fourth, our study had a cross-sectional design; therefore, we could not deduce from our findings any causal relationship between diagnosis effect modulated by the gene and WM microstructural change in the PHC. Finally, we did not include psychosocial factors such as childhood adversity or stressful life events as dependent variables in our analyses. Numerous studies have consistently suggested that these factors influence WM integrity in MDD patients [40]. We hope that future imaging genetic studies of this gene could include these psychosocial factors, to allow for more comprehensive and fundamental research.

In conclusion, we observed risk allele of *SLC6A15* gene rs1545843 influence on WM integrity of the PHC in MDD patients, which is known to play an important role in the neural circuit involved in emotion processing. We believe that this finding will help expand our knowledge of the neurobiological basis of the development of MDD by elucidating the genetic effect of a novel candidate gene and its influence on structural brain change in MDD patients.

## Supporting Information

S1 Table The list of the 42 white matter tracts in the whole-brain analysis. (DOCX)

S2 Table The differences in fractional anisotropy values in the whole-brain white matter tracts among the groups determined by genotype and diagnosis. (DOCX)
