# Supplementary material for: Effects of a Polymorphism of the Neuronal Amino Acid Transporter SLC6A15 Gene on Structural Integrity of White Matter Tracts in Major Depressive Disorder
Source: PLoS One. 2016 Oct 10;11(10):e0164301. doi: 10.1371/journal.pone.0164301 (PMC5056691; doi:10.1371/journal.pone.0164301)
Supplement: S1 Table — (DOCX) [file pone.0164301.s001.docx]

S1 Table. The list of the 42 white matter tracts in the whole-brain analysis

| **Name** | **Abbreviation** |
| --- | --- |
| Anterior corona radiata, left | L ACR |
| Anterior corona radiata, right | R ACR |
| Anterior limb of internal capsule, left | L ALIC |
| Anterior limb of internal capsule, right | R ALIC |
| Body of corpus callosum | BCC |
| Cerebral peduncle, left | L CP |
| Cerebral peduncle, right | R CP |
| Cingulum (cingulate gyrus), left | L CGC |
| Cingulum (cingulate gyrus), right | R CGC |
| Cingulum (hippocampus), left | L PHC |
| Cingulum (hippocampus), right | R PHC |
| Corticospinal tract, left | L CST |
| Corticospinal tract, right | R CST |
| External capsule, left | L EC |
| External capsule, right | R EC |
| Fornix (column and body of fornix) | FX |
| Fornix (cres) / Stria terminalis, left | L FX-ST |
| Fornix (cres) / Stria terminalis, right | R FX-ST |
| Genu of corpus callosum | GCC |
| Inferior cerebellar peduncle, left | L ICP |
| Inferior cerebellar peduncle, right | L ICP |
| Inferior fronto-occipital fasciculus, left | L IFO |
| Inferior fronto-occipital fasciculus, right | R IFO |
| Posterior corona radiata, left | L PCR |
| Posterior corona radiata, right | R PCR |
| Posterior limb of internal capsule, left | L PLIC |
| Posterior limb of internal capsule, right | R PLIC |
| Posterior thalamic radiation (include optic radiation), left | L PTR |
| Posterior thalamic radiation (include optic radiation), right | R PTR |
| Retrolenticular part of internal capsule, left | L RLIC |
| Retrolenticular part of internal capsule, right | R RLIC |
| Sagittal stratum (include inferior longitudinal fasciculus and IFO), left | L SS |
| Sagittal stratum (include inferior longitudinal fasciculus and IFO), right | R SS |
| Splenium of corpus callosum | SCC |
| Superior corona radiata, left | L SCR |
| Superior corona radiata, right | R SCR |
| Superior fronto-occipital fasciculus, left | L SFO |
| Superior fronto-occipital fasciculus, right | R SFO |
| Superior longitudinal fasciculus, left | L SLF |
| Superior longitudinal fasciculus, right | R SLF |
| Uncinate fasciculus, left | L UF |
| Uncinate fasciculus, right | R UF |

The white matter tracts were derived from the Johns Hopkins University (JHU) white matter tractography atlas implemented in the FMRIB Diffusion Toolbox (FDT) software.

L, left hemisphere; R, right hemisphere.
