# Supplementary material for: Effects of a Polymorphism of the Neuronal Amino Acid Transporter SLC6A15 Gene on Structural Integrity of White Matter Tracts in Major Depressive Disorder
Source: PLoS One. 2016 Oct 10;11(10):e0164301. doi: 10.1371/journal.pone.0164301 (PMC5056691; doi:10.1371/journal.pone.0164301)
Supplement: S2 Table — (DOCX) [file pone.0164301.s002.docx]

S2 Table. The differences in fractional anisotropy values in the whole-brain white matter tracts among the groups determined by genotype and diagnosis.

| **WM tracts** | **MDD vs. HC** | |  | **AG/GG vs. AA** | | **Diagnosis x Genotype interaction** | |
| --- | --- | --- | --- | --- | --- | --- | --- |
|  | **F** | **p** |  | **F** | **p** | **F** | **p** |
| L ACR | 0.981 | 0.324 |  | 0.022 | 0.882 | 1.839 | 0.177 |
| R ACR | 2.489 | 0.117 |  | 0.303 | 0.583 | 0.675 | 0.413 |
| L ALIC | 8.786 | 0.004* | MDD > HC | 0.294 | 0.588 | 1.623 | 0.205 |
| R ALIC | 1.298 | 0.256 |  | 0.549 | 0.460 | 3.863 | 0.051 |
| BCC | 10.874 | 0.001* | MDD < HC | 0.009 | 0.925 | 0.840 | 0.361 |
| L CP | 0.015 | 0.902 |  | 0.001 | 0.975 | 1.628 | 0.204 |
| R CP | 0.152 | 0.697 |  | 0.117 | 0.733 | 0.787 | 0.377 |
| L CGC | 0.156 | 0.694 |  | 0.052 | 0.820 | 0.143 | 0.706 |
| R CGC | <0.001 | 0.990 |  | 0.105 | 0.747 | 0.252 | 0.616 |
| L PHC | 8.698 | 0.004* | MDD < HC | 0.127 | 0.722 | 0.221 | 0.639 |
| R PHC | 1.323 | 0.252 |  | 0.140 | 0.709 | 0.026 | 0.873 |
| L CST | 0.440 | 0.508 |  | 0.593 | 0.443 | 0.013 | 0.908 |
| R CST | 1.865 | 0.174 |  | 0.042 | 0.839 | 0.786 | 0.377 |
| L EC | 4.497 | 0.036 |  | 0.177 | 0.675 | 0.142 | 0.707 |
| R EC | 4.716 | 0.032 |  | 0.327 | 0.568 | 0.389 | 0.534 |
| FX | 2.102 | 0.149 |  | 2.612 | 0.108 | 0.133 | 0.716 |
| L FX-ST | 0.063 | 0.803 |  | 0.848 | 0.359 | 0.114 | 0.736 |
| R FX-ST | 0.834 | 0.363 |  | 1.349 | 0.247 | 1.571 | 0.212 |
| GCC | 2.764 | 0.099 |  | 0.528 | 0.469 | 0.198 | 0.657 |
| L ICP | 6.760 | 0.010 |  | 0.192 | 0.662 | 2.058 | 0.154 |
| R ICP | 2.136 | 0.146 |  | 0.462 | 0.498 | 3.494 | 0.064 |
| L IFO | 0.411 | 0.523 |  | 0.862 | 0.355 | 0.152 | 0.698 |
| R IFO | 1.349 | 0.247 |  | 0.019 | 0.891 | 6.870 | 0.010 |
| L PCR | 0.021 | 0.886 |  | 0.048 | 0.826 | 1.875 | 0.173 |
| R PCR | 0.347 | 0.557 |  | 0.221 | 0.639 | 0.974 | 0.325 |
| L PLIC | 7.803 | 0.006 |  | 0.152 | 0.697 | 2.372 | 0.126 |
| R PLIC | 11.241 | 0.001* | MDD > HC | 0.761 | 0.384 | 3.144 | 0.078 |
| L PTR | 4.031 | 0.047 |  | 0.002 | 0.963 | 3.003 | 0.085 |
| R PTR | 1.100 | 0.296 |  | 0.003 | 0.960 | 0.485 | 0.487 |
| L RLIC | 1.277 | 0.260 |  | 0.010 | 0.921 | 0.131 | 0.717 |
| R RLIC | 2.067 | 0.153 |  | 0.003 | 0.956 | 1.087 | 0.299 |
| L SS | 1.303 | 0.256 |  | 0.087 | 0.769 | 1.944 | 0.165 |
| R SS | 0.016 | 0.899 |  | 0.131 | 0.717 | 0.039 | 0.844 |
| SCC | 2.223 | 0.138 |  | 0.567 | 0.453 | 2.22 | 0.138 |
| L SCR | 2.213 | 0.139 |  | 0.239 | 0.626 | 0.376 | 0.541 |
| R SCR | 3.186 | 0.076 |  | 0.209 | 0.648 | 0.339 | 0.561 |
| L SFO | 2.930 | 0.089 |  | 1.060 | 0.305 | 0.045 | 0.832 |
| R SFO | 1.217 | 0.272 |  | 0.161 | 0.688 | 1.294 | 0.257 |
| L SLF | 1.307 | 0.255 |  | 0.417 | 0.519 | 3.329 | 0.070 |
| R SLF | 1.519 | 0.220 |  | 0.398 | 0.529 | 0.41 | 0.523 |
| L UF | 0.554 | 0.458 |  | 3.705 | 0.056 | 3.662 | 0.058 |
| R UF | 0.113 | 0.737 |  | 0.136 | 0.713 | 0.658 | 0.419 |

The F and uncorrected-P values were obtained using two-way analysis of covariance (ANCOVA) adjusted for age, sex, and total intracranial cavity volume as covariates.

FDR was applied in the comparisons of the 42 white matter tracts in both hemispheres, q < 0.05.

*Regions that remained significant after FDR correction are marked with an asterisk.

MDD, major depressive disorder; HC, healthy controls; Diagnosis × Genotype interaction, the diagnosis (patients with MDD vs. healthy controls)-by-genotype (GG vs. AA/AG) interaction.
